# Social norms explain prioritization of climate policy

**DOI:** 10.1007/s10584-022-03396-x

**Published:** 2022-07-18

**Authors:** Jennifer C. Cole, Phillip J. Ehret, David K. Sherman, Leaf Van Boven

**Affiliations:** 1 Department of Psychology and Neuroscience, University of Colorado Boulder, Boulder, USA; 2 Climate Change Research Network, Vanderbilt University, PMB 351805, 2301, Vanderbilt Place 37235-1805, Nashville, TN USA; 3 See Change Institute, Venice, USA; 4 Department of Psychology and Brain Sciences, University of California, Santa Barbara, USA

**Keywords:** Social norms, Climate policy, Policy support, Policy prioritization

## Abstract

Most people in the United States recognize the reality of climate change and are concerned about its consequences, yet climate change is a low priority relative to other policy issues. Recognizing that belief in climate change does not necessarily translate to prioritizing climate policy, we examine psychological factors that may boost or inhibit prioritization. We hypothesized that perceived social norms from people’s own political party influence their climate policy prioritization beyond their personal belief in climate change. In Study 1, a large, diverse sample of Democratic and Republican participants (*N* = 887) reported their prioritization of climate policy relative to other issues. Participants’ perceptions of their political ingroup’s social norms about climate policy prioritization were the strongest predictor of personal climate policy prioritization—stronger even than participants’ belief in climate change, political orientation, environmental identity, and environmental values. Perceptions of political outgroup norms did not predict prioritization. In Study 2 (*N* = 217), we experimentally manipulated Democratic and Republican descriptive norms of climate policy prioritization. Participants’ prioritization of climate policy was highest when both the political ingroup and the outgroup prioritized climate policy. Ingroup norms had a strong influence on personal policy prioritization whereas outgroup norms did not. These findings demonstrate that, beyond personal beliefs and other individual differences, ingroup social norms shape the public’s prioritization of climate change as a policy issue.

**Supplementary Information:**

The online version contains supplementary material available at 10.1007/s10584-022-03396-x.

## Introduction

The Executive Order on Tackling the Climate Crisis at Home and Abroad from the White House of the United States (White House 2021) declared, “The United States and the world face a profound climate crisis. We have a narrow window in time to pursue action….Domestic action must go hand in hand with international leadership by the United States that is aimed at significantly enhancing global action.” Despite the declared urgency, climate policy remains a low priority relative to other policy issues such as creating jobs and improving education (Funk and Kennedy 2020; Pew Research Center 2017, 2021). The relative deprioritization of climate policy is true among the general public and is pronounced among Republicans (Kennedy and Johnson 2020). Deprioritization has also been especially pronounced since the emergence of the COVID-19 pandemic (Pew Research Center 2021). In one poll, respondents ranked climate change fifteenth among nineteen policy issues (Pew Research Center 2021).

What influences the public’s prioritization of climate change relative to other policy issues? Belief in climate change is one important predictor (Hornsey et al. 2016). Yet most people, both Democrats and Republicans, do believe in climate change (Marlon et al. 2020), even as they deprioritize climate policy. What other factors explain climate policy prioritization?

We hypothesized that perceived social norms of how much other people prioritize climate policy influence people’s personal prioritization of climate policy. We further hypothesized that within the United States, where climate policy is sharply polarized (Van Boven et al. 2018), perceived norms among people’s own political party positively influence their policy prioritization more than perceived norms among people’s political outgroup negatively influence policy prioritization. We derived these hypotheses from social psychological research emphasizing the categorization of the self and others into ingroups and outgroups (Tajfel and Turner, 1979; Turner et al. 1987), and from research on the influence of social norms on attitudes (Miller and Prentice 2016; Tankard and Paluck 2016). Previous research on attitudes toward climate change focused on political orientation, environmental identities, environmental values, and other individual differences (e.g., Campbell and Kay 2014; Fielding et al. 2012; McCright and Dunlap 2011). We hypothesize that perceived social norms influence climate policy prioritization over and above these other factors.

### Prioritization versus support of climate policy

The necessity of prioritizing climate policy relative to other issues results from governments’ limited resources and the necessary tradeoffs of budgets, time, and capacity (Jones and Baumgartner 2005; Natchez and Bupp 1973). Public opinion influences a range of governmental processes such as elections (Stimson et al. 1995) and policy design and implementation (Budge and Hofferbert 1990; Burstein 2003; Jones and Baumgartner 2005). Policymakers must sort through a surplus of information in the political environment when deciding which issues and policies to prioritize. Because of the correspondence between the public’s prioritization of issues and policymakers’ prioritization of issues, strong signals that the public prioritizes an issue could influence policymakers’ attention to the issue and to solutions they support (Jones and Baumgartner 2005).

The seeming contradiction between many people in the United States and elsewhere prioritizing other issues over climate change while believing that climate change poses a global threat was well characterized by Former U.S. Representative Mickey Edwards (Republican, OK, 1977–1993; Van Boven et al. 2018):

[People] say, “Well, we do understand that there’s a climate problem. We do understand that it is harmful, but now [policymakers] are hearing directly, including from the people advocating, what the other side of it is. This is not all benefit and no cost. … [W]e’re looking at weighing the cost versus the benefit.”

Tradeoffs such as these between addressing climate change and addressing other issues can dissuade people from prioritizing climate policy. In 2020, 72% of Americans recognized that climate change is happening and 75% supported the regulation of carbon dioxide emissions (Marlon et al. 2020). Yet only 38% considered global warming a top priority in comparison to other policy issues (Pew Research Center 2021). Instead, people prioritized strengthening the economy and addressing the COVID-19 pandemic (Pew Research Center 2021), despite the fact that climate change is more of a looming existential threat. If people recognize the reality of climate change and support climate policies, why do they not prioritize climate change relative to other policy issues?

### Social norms

We suggest that perceived social norms influence climate policy prioritization. *Social norms* comprise perceptions of typical or desired attitudes and behaviors within social groups (Sherif 1936), shaping people’s construal of what is common, correct, and valued (Miller and Prentice 1996). People are motivated to align their attitudes, beliefs, and behaviors with the social norms of their groups (Hogg and Reid 2006; Miller and Prentice 2016; Tankard and Paluck 2016). Norms influence political attitudes and policy preferences (Mutz 1998; Sherman et al. 2021) and are likely to influence people’s prioritization of climate policy.

Previous work provides indirect evidence that social norms influence attitudes toward climate policy—although not climate policy prioritization—by experimentally manipulating political elites’ stances toward climate policy (Ehret et al. 2018; Van Boven et al. 2018). In that research, Democratic and Republican participants supported climate policies that were backed by ingroup political elites more than the same policies backed by outgroup political elites, conceptually replicating research on how cues from political elites influence political attitudes (Cohen 2003; Zaller 1992; Van Boven and Sherman 2021). Elite stances influenced people’s perceived social norms; people expected social norms to align with elite stances (Ehret et al. 2018; Van Boven et al. 2018). Elites thus convey what social norms about climate change attitudes are within their political parties, which then influences climate policy attitudes.

Other work directly examined how social norms influence attitudes toward environmental policy (De Groot and Schuitema 2012; Rinscheid et al. 2021). In one study, informing people in the UK that their peers either supported or opposed environmental and climate policies regarding car use and littering influenced personal support of these policies (De Groot and Schuitema, 2021). Another study manipulated supposed peer use of sustainable transportation and support or opposition of carbon capture and sequestration policy, and asked participants to rate their likelihood to vote for those policies (Rinscheid et al. 2021). Negative social norms (of not using sustainable transportation) but not positive social norms (of using sustainable transportation) influenced people’s likelihood to vote for sustainable transportation policies; social norms did not influence people’s support for carbon capture and sequestration. Our studies build on previous research by experimentally manipulating norms specific to political parties, rather than norms among the general public, and by comparing norms from political ingroups and outgroups.

Social psychological research suggests that ingroup norms influence attitudes and behaviors differently than do outgroup norms. People seek to align with ingroups and differentiate from outgroups (Berger and Heath 2008; Chan et al. 2012; Iyengar et al. 1999; Mummendey and Wenzel 1999). Ingroup norms provide cues that help group members cohere around a shared set of attitudes, beliefs, and behaviors (e.g., “Democrats like us prioritize climate policy…”). Outgroup norms provide a contrast that defines group boundaries (e.g., “…unlike our Republican opponents who do not prioritize climate policy”).

Some theorists argue that ingroup influence is stronger than outgroup influence (Allport, 1954; Brewer 1999), whereas other theorists emphasize the influence of outgroups (Tajfel and Turner, 1979). Research on identity signaling has demonstrated that people’s environmental actions in particular are often motivated by a desire to contrast their actions with an unwanted social category, such as conservatives who avoid buying energy-efficient lightbulbs when they are labeled as environmentally friendly (Gromet et al. 2015; see also Brick et al. 2017). However, in the studies summarized earlier (Ehret et al. 2018; Van Boven et al. 2018), perceived ingroup norms communicated through experimentally manipulated elite cues more strongly predicted participants’ climate policy stances than did perceived outgroup norms (Ehret et al. 2018; Van Boven et al. 2018). In those studies, norms were measured and their relationship with personal policy support was analyzed correlationally. In highly polarized contexts such as climate policy in the United States, perceptions of political ingroups and outgroups are negatively correlated with each other (Mullen et al. 1992; Riketta 2005; Westfall et al. 2015). It is therefore difficult yet important to tease apart the effects of ingroup norms and outgroup norms unless they are separately measured and manipulated.

### Ideology, identities, and values

We examine the effect of social norms on policy prioritization relative to other relevant psychological factors. Previous research demonstrated individual difference characteristics such as personal ideologies and identities as important predictors of attitudes toward climate policy (e.g., Gatersleben et al. 2014; Hornsey et al. 2016). Our work extends this by examining how climate policy prioritization is influenced by social factors such as perceived social norms, over and above individual difference characteristics. This reflects a need for more research on group processes in environmental psychology (Masson and Fritsche 2021; Pearson et al. 2016). We considered the following individual differences in our studies.

*Political ideology* encompasses liberal versus conservative values about the ideal structure of society, how this structure should be achieved, and the role of government in achieving this structure. It incorporates values on social, economic, and environmental issues (Gerring 1997; Jost et al. 2008, 2009). Liberal ideology is associated with higher belief in climate change and support for climate policy than is conservative ideology (Campbell and Kay 2014; Fielding et al. 2012; Hornsey et al. 2016; Kahan et al. 2011; Ziegler 2017). Relatedly but distinct, *partisan identification* as a Democrat or Republican is a social identity (Greene 1999). In the United States, Democrats are more liberal, hold stronger environmental values, are more certain of climate change, and are more supportive of environmental policy than are Republicans (DeNicola and Subramaniam 2014; Dunlap and McCright 2008; Kahan 2012; McCright and Dunlap 2011). Because ideology and partisan identification are strongly correlated at present in the United States (Arnold 2009; Jacobs et al. 2019; Klein 2020), we aggregate measures of these two constructs in the present research.

*Environmental identity* reflects a social identity as an environmentalist (Brick et al. 2017; Clayton 2003; Gatersleben et al. 2014) and is a strong predictor of environmental attitudes and beliefs (Brick et al. 2017; Carfora et al. 2017; Gatersleben et al. 2014; Van der Werff et al. 2013; Whitmarsh and O’Neill 2010). *Environmental values* reflect concern about the environment and views about how humans relate to nature (Arcury 1986; Dunlap 2008; Dunlap and Van Liere 1978; Dunlap et al. 2000). Values are correlated with knowledge about environmental issues (Arcury 1986), support for environmental policy (Poortinga et al. 2002, 2004), and pro-environmental behaviors (Clark et al. 2003). Environmental identity and values are distinct constructs that are moderately correlated (Gatersleben et al. 2014; Maki et al. 2019), so we examine them separately in the present research.

We expected to conceptually replicate previous findings that ideology, identities, and values correlate with climate policy prioritization just as they correlate with belief in climate change and support for climate policy. We also expected perceived social norms to predict climate policy prioritization over and above these factors.

### Overview of present studies

In Study 1, we surveyed a large, diverse national sample of participants in the United States. We examined whether people’s perceptions of their political ingroup’s prioritization of climate policy would predict their personal policy prioritization, and whether they would do so over and above people’s belief in climate change, political orientation, environmental identity, environmental values, perceptions of outgroup prioritization, and demographic characteristics. In Study 2, we experimentally manipulated social norms of climate policy prioritization of Democrats and Republicans and compared the influence of social norms from one’s political ingroup and political outgroup. We explored whether personal prioritization of climate policy would be highest on average, across the full sample, when both parties’ norms supported prioritization of climate policy, a finding that would imply the potential for bipartisan policy prioritization outside of the lab.

The University of Colorado Institutional Review Board approved the studies. Data, full materials, analysis code, and results are available on the Open Science Framework (OSF): https://osf.io/mf945

## Study 1

Participants reported their prioritization of climate policy, perceived descriptive social norms about Democratic and Republican climate policy prioritization and belief in climate change, and individual difference characteristics. We expected perceived ingroup norms to predict participants’ prioritization of climate policy over and above outgroup norms, norms of belief in climate change within the political parties, personal belief in climate change, and individual difference characteristics. We explored whether perceived ingroup norms would be a stronger predictor than these other factors.

### Participants

Participants (*N* = 1.065) were recruited by the Denver-based market research firm ROI Rocket using stratified sampling procedures based on benchmarks from the U.S. Census and Pew Research Center (see ESM Table 1 for full demographic information). The sample size was determined a priori by the project budget. A sensitivity analysis indicated sufficient power to detect small effect sizes of Cohen’s *d* = 0.19 for regression coefficients in our largest regression model. Data were collected between 27 October and 8 November 2016. Portions of these data were presented in Van Boven et al. (2018) to examine other hypotheses. Participants were compensated monetarily for their participation per ROI Rocket’s standards. We excluded political independents from our analyses because our hypotheses about ingroup and outgroup norms could only apply to participants who identified with a political party. The analytic sample included 463 Democrats and 424 Republicans (total *N* = 887). The sample varied in age (*M* = 45.93, *SD* = 16.85), gender (50.23% female, 49.21% male, and 0.56% other), and ethnicity (3.95% Asian or Asian-American, 11.64% Black or African-American, 14.92% Hispanic or Latino-American, 0.45% Native American, 0.23% Native Pacific Islander, 68.25% White, and 0.56% other race) Table 1.

### Measures

#### Climate policy prioritization and belief in climate change

For our key outcome, we measured prioritization of climate policy. We asked participants how they thought the president and Congress should prioritize climate change (“Dealing with climate change and global warming”) relative to other policy issues (drawing from the list of policy issues used by Pew Research Center 2017). Participants rated the importance of 19 policy issues, randomly ordered, for the next president and Congress on a 4-point scale labeled: *should not be done* (+ 1), *not too important* (+ 2), *important but lower priority* (+ 3), *top priority* (+ 4). Other policy issues included, for example, “strengthening the nation’s economy,” “improving the educational system,” “dealing with issues of illegal immigration,” and “strengthening the U.S. Military.” Participants could mark as many issues as they wanted as high or low priority.

We calculated *z*-scores to represent participants’ prioritization of climate policy relative to other policy issues using the following formula:

$$z_{\mathrm{climate}\;\mathrm{policy}}=\frac{\mathrm{respondent}\;\mathrm{climate}\;\mathrm{policy}\;\mathrm{rating}-\mathrm{mean}\;\mathrm{rating}\;\mathrm{of}\;\mathrm{issues}\;\mathrm{for}\;\mathrm{respondent}}{\mathrm{SD}\;\mathrm{of}\;\mathrm{rating}\;\mathrm{of}\;\mathrm{issues}\;\mathrm{for}\;\mathrm{respondent}}$$

Each participant’s *z*-score indicated how highly climate policy was prioritized relative to their mean prioritization of issues. As such, this scoring method captures issue prioritization in the context of each individual’s beliefs about the scope of government action in general.

We measured belief in climate change by asking participants to read a short description of climate change and to report their agreement on a 7-point scale from *strongly disagree* (– 3) to *strongly agree* (+ 3) with four statements: climate change is happening; climate change poses a risk to human health, safety, and prosperity; human activity is largely responsible for recent climate change; and reducing greenhouse gas emissions will reduce global warming (Van Boven et al. 2018). We averaged the four ratings into a composite index (α = 0.92).

#### Perceived social norms about policy prioritization and belief in climate change

We measured perceived social norms about policy prioritization by asking participants how they thought the average Democrat and Republican, in randomized order, would prioritize the same list of policy issues for which participants rated their own prioritization. We calculated *z*-scores for perceptions of Democratic and Republican prioritization of climate policy just as for personal policy prioritization. We measured perceived social norms about belief in climate change by asking participants how the average Democrat and Republican, in randomized order, would respond to the same items about belief in climate change on which they reported their personal belief (α_Dem_ = 0.95, α_Rep_ = 0.96).

For analysis, we coded predictors of ingroup norms of policy prioritization and belief in climate change that consisted of Democrats’ perceptions of Democrats’ attitudes and Republicans’ perceptions of Republicans’ attitudes. We coded predictors of outgroup norms of policy prioritization and belief in climate change that consisted of Democrats’ perceptions of Republicans’ attitudes and Republicans’ perceptions of Democrats’ attitudes.

#### Measures of ideology, identities, and values

Participants rated their ideology on political, economic, and social issues on three separate 7-point scales from *very liberal* (– 3) to *very conservative* (+ 3). Although conceptually distinct (Jost et al. 2009), these three dimensions of ideology were strongly correlated (α = 0.94) and we averaged them into a composite index. We measured partisan identification with either the Democratic or Republican political party using branching questions from the American National Election Study, yielding a 7-point continuous measure from *strong Democrat* (– 3) to *strong Republican* (+ 3). We averaged political ideology and partisan identification scores (*r* = 0.57, *p* < .001) to create a composite 7-point measure of political orientation.

Participants reported their environmental identity by indicating their agreement on a 7-point scale from *strongly disagree* (– 3) to *strongly agree* (+ 3) with two statements: “I am concerned with environmental issues,” and “I am an environmentalist” (*r* = 0.73, *p* < .001). We measured environmental values with the New Ecological Paradigm (NEP; Dunlap et al. 2000), including agreement a 5-point scale from *strongly disagree* (– 2) to *strongly agree* (+ 2) with 15 items such as “humans are seriously abusing the environment.” We averaged these into a composite index (α = 0.82).

#### Demographics

Participants reported their gender as male (– 1), female (+ 1), or other. The five participants (0.56%) who reported their gender as “other” were not coded as part of this predictor due to the small sample size. Participants reported their highest level of educational attainment, coded for analysis on a 7-point scale from *grammar school* (+ 1) to *doctoral degree* (PhD) or *professional degree* (MD, JD, etc.; + 7). They reported their income on a 9-point scale from *under $10,000* (+ 1) to *over $150,000* (+ 9). They also provided their age. Finally, participants reported their race or ethnicity as Asian or Asian-American, Black or African-American, Hispanic or Latino-American, Native American, Native Pacific Islander, White or Caucasian-American, or Other. We created three dummy codes which indicated identification as either Black or Hispanic (the two largest minority groups in the United States; U.S. Census Bureau 2019, and in our sample), or another minority group (including Asian, Native American, or Native Pacific Islander), with White as the reference group.

### Results

All available data were included for descriptive statistics. We used listwise deletion in regression models to include all participants who responded to all relevant variables (sample sizes provided in Tables 1, 2 and 3).

#### Descriptive statistics and zero-order correlations

Table 1 summarizes descriptive statistics and zero-order correlations between psychological measures. Democrats prioritized climate policy (*M* = 0.03, *SD* = 1.01) higher than did Republicans (*M* = – 0.97, *SD* = 1.23; *t*(803) = 12.59, *p* < .001). They also believed in climate change (*M* = 1.71, *SD* = 1.32) more strongly than did Republicans (*M* = 0.65, *SD* = 1.52; *t*(882) = 13.17, *p* < .001), although both groups were above the neutral midpoint. Belief in climate change was correlated with prioritization of climate policy (*r*(803) = 0.57, *p* < .001).

The correlations of other measures with belief in climate change replicated previous research. Belief in climate change was correlated with being liberal and Democratic, higher environmental values, and stronger environmental identity (Campbell and Kay 2014; Fielding et al. 2012; Fielding and Hornsey 2016; Hornsey et al. 2016; Van Boven et al. 2018). Climate policy prioritization was similarly correlated with all these measures.

**Table 1:** Study 1 descriptive statistics and correlations

	A	B	C	D	E	F	G	H	I
*Mean*	– 0.45	– 0.47	– 0.02	1.31	1.20	0.67	0.09	0.61	0.39
*SD*	1.22	1.17	1.17	1.49	1.57	1.93	1.81	1.64	0.61
*N*	805	746	774	884	882	883	887	887	886
A.Participant climate policy prioritization									
B.Ingroup climate policy prioritization	.56								
C.Outgroup climate policy prioritization	– .36	– .45							
D.Participant belief in climate change	.57	.47	– .40						
E.Ingroup belief in climate change	.47	.50	– .43	.77					
F.Outgroup belief in climate change	– .30	– .35	.52	– .20	– .24				
G.Political orientation	– .47	– .53	.49	– .45	– .47	.42			
H.Environmental identity	.44	.33	– .26	.60	.50	– .12	– .28		
I.Environmental values	.45	.32	– .33	.62	.46	– .35	– .36	.41	

Variables represented in the columns correspond to the variables in the rows by letter. Cells represent correlations between the variables in each row/column pair. All correlations are significant at *p* < .001. Policy prioritization measures are *z*-scores, comparing prioritization of climate policy to prioritization of 18 other policy issues. Negative *z*-scores indicate prioritization of climate policy below the mean prioritization of all issues. Belief in climate change, political orientation, and environmental identity are coded on 7-point Likert scales. Higher scores on political orientation indicate being more conservative and Republican. Environmental values are coded on a 5-point Likert scale

#### Ideology, identities, and values predict climate policy prioritization

We first regressed participants’ prioritization of climate policy on political orientation, environmental identity, and environmental values. These factors all predicted prioritization of climate change, controlling for demographics (adjusted *R*^2^ = 0.42, Model 1 in Table 2), conceptually replicating previous research.

**Table 2:** Study 1 regression models predicting climate policy prioritization by norms and individual differences

	Model 1			Model 2		
	*b*	*SE*	η_*p*_^2^	*b*	*SE*	η_*p*_^2^
Intercept	– 0.49***	0.04	0.15	– 0.35***	0.05	0.09
*Social norms*						
Ingroup climate policy prioritization				0.29***	0.04	0.08
Outgroup climate policy prioritization				0.02	0.04	< 0.01
Ingroup belief in climate change				– 0.02	0.03	< 0.01
Outgroup belief in climate change				– 0.03	0.02	< 0.01
*Ideology, identity, and values*						
Personal belief in climate change	0.24***	0.04	0.06	0.20***	0.04	0.04
Political orientation	– 0.10***	0.03	0.02	– 0.08*	0.03	0.01
Environmental identity	0.10***	0.03	0.02	0.09**	0.03	0.02
Environmental values (NEP)	0.25**	0.08	0.02	0.27***	0.08	0.02
*Demographics*						
Age	– 0.01***	< 0.01	0.02	– 0.01**	< 0.01	0.01
Income	0.03	0.02	0.01	0.03	0.02	0.01
Gender	– 0.03	0.04	< 0.01	– 0.03	0.04	< 0.01
Education	0.03	0.03	< 0.01	0.02	0.03	< 0.01
Black	0.05	0.12	< 0.01	– 0.09	0.12	< 0.01
Hispanic	0.18	0.10	< 0.01	0.25*	0.11	0.01
Other Minority	0.41*	0.16	0.01	0.32*	0.16	0.01
Adjusted *R*^2^		.42			.51	

*Note.* *** indicates *p* < .001; ** indicates *p* < .01; * indicates *p* < .05. Sample sizes vary due to missing data (Model 1: *N* = 820; Model 2: *N* = 664). Coefficients are unstandardized. Belief in climate change, environmental identity, environmental values, age, income, and education are continuous. Higher scores on political orientation indicate being more conservative and Republican. Gender is coded as male (– 1) and female (+ 1). Black, Hispanic or Latino, and Other Minority are dummy coded with White as the reference group

#### Perceived social norms predict climate policy prioritization

We next tested our main hypotheses regarding perceived social norms of participants’ political ingroups and outgroups. Perceived norms within the ingroup and outgroup were negatively correlated with each other for both climate policy prioritization (*r* = – 0.45, *p* < .001) and belief in climate change (*r* = – 0.24, *p* < .001).

We added participants’ estimates of ingroup and outgroup climate policy prioritization and their estimates of ingroup and outgroup belief in climate change to the previous model, which significantly increased model fit (to adjusted *R*^2^ = 0.51; test of increase: *F*(4, 649) = 15.77, *p* < .001; Model 2 in Table 2). As predicted, perceived ingroup policy prioritization predicted participants’ personal climate policy prioritization (*b* = 0.29, *SE* = 0.04, η_*p*_^2^ = 0.08, *p* < .001). The effect of perceived ingroup norms was significantly larger (*F*(1, 649) = 28.45, *p* < .001) than that of perceived outgroup norms, which was not significant (*b* = 0.02, *SE* = 0.04, η_*p*_^2^ < 0.01, *p* = .601).

Adding social norms to the model did not substantively change the patterns of other predictors; yet, perceived ingroup policy prioritization was a stronger predictor (η_*p*_^2^ = 0.08) of policy prioritization than these other predictors, even stronger than participants’ personal belief in climate change (η_*p*_^2^ = 0.04). And while perceived ingroup norms of policy prioritization predicted personal prioritization, perceived ingroup belief in climate change did not (*b* = – 0.02, *SE* = 0.03, η_*p*_^2^ < 0.01, *p* = .502), indicating that belief in climate change and prioritization of climate policy are two distinct constructs. This also demonstrates that social norms about climate policy attitudes are distinct from climate change attitudes generally. These results provide support for the importance of individuals’ perceptions of ingroup policy prioritization norms to their personal prioritization of climate policy.^1^

### Discussion

Study 1 demonstrated that perceived social norms about ingroup prioritization of climate policy strongly predict personal prioritization of climate policy, more so than perceived outgroup norms, belief in climate change, political orientation, environmental identity, and environmental values. Because these results are correlational, however, they might be explained by some other unmeasured factors, or by reverse causality—that people project their own climate policy prioritization onto their political ingroup (Robbins and Krueger 2005; Ross et al. 1977). Given the causal ambiguity inherent in correlational findings, we next sought experimental evidence that ingroup norms influence policy prioritization to establish causality.

## Study 2

We conducted an experiment to test whether ingroup norms influence personal prioritization of climate policy, and we compared the influences of ingroup norms and outgroup norms. A negative effect of outgroup norms would be practically meaningful because, in the United States, bipartisan prioritization of climate policy outside of the lab would be unattainable if people do not prioritize an issue that the other party prioritizes. We included a condition in which both ingroup and outgroup norms prioritized climate policy to examine this possibility. Given the practical significance of this condition, we also exploratorily compared personal prioritization in this bipartisan condition to average personal prioritization across the other conditions to examine whether bipartisan norms of prioritization would yield the highest levels of personal prioritization.

To operationalize ingroup and outgroup norms, we included orthogonal manipulations of Democratic and Republican descriptive social norms. Participants indicated their own prioritization of climate policy after reading an infographic depicting prioritization of climate policy among ordinary Democrats and Republicans. Including participants’ partisan identification, this translated into a 2 (Ingroup norms: prioritize or do not prioritize) × 2 (Outgroup norms: agree with ingroup or oppose ingroup) × 2 (Partisan identification: Democrat or Republican) factorial design.

### Participants

Participants (*N* = 217) from Amazon Mechanical Turk received $1.00 in exchange for completing the study. We collected 284 responses. Our final analysis sample included 217 responses after dropping political independents (*n* = 11) and excluding participants in a control condition that was not relevant to hypotheses of this paper (*n* = 56).^2^ Within our 2 × 2 × 2 factorial design, cell sizes ranged from 18 to 22 for Republicans across norms conditions and 33 to 36 for Democrats across norms conditions (ESM Table 3). A sensitivity power analysis showed that this sample size was sufficient to detect a small to medium-sized main or interaction effect of Cohen’s *d* of 0.38. For analysis of the effects of ingroup and outgroup norms, we collapsed across partisan identification to examine main effects for the whole sample. In this design, the sample is more appropriately powered to detect main effects of norms and is less well powered to detect interactions between norms and partisan identification. Thus, our main conclusions encompass the power of social norms across both political parties. We encourage future work to examine differences in effects between parties.

We collected data on 11 and 12 October 2019. The sample varied in age (*M* = 38.30, *SD* = 11.36), gender (50.23% female, 48.85% male, and 0.92% other), and ethnicity (12.44% Asian or Asian-American, 5.99% Black or African-American, 7.37% Hispanic or Latino-American, 1.38% Native American, 70.97% White, and 1.84% other race).

### Procedure

Participants reported their partisan identification and personal belief in climate change, randomly ordered, using the same measures as in Study 1. The sample included 137 Democrats (63.13%) and 80 Republicans (36.87%).

**Fig. 1:**
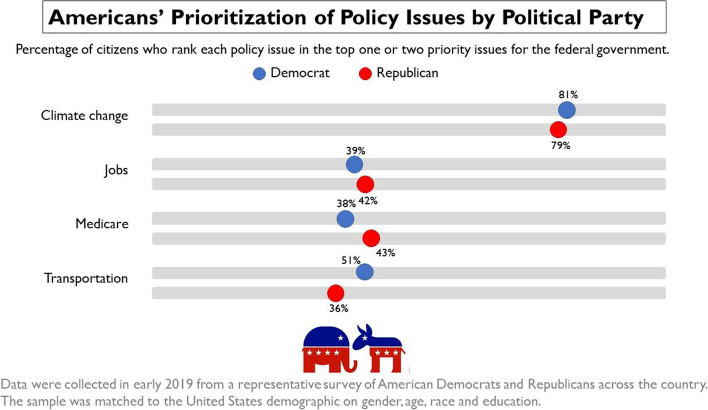
An example infographic displaying normative information about prioritization of climate policy by party in Study 2, depicting the condition where both parties prioritize climate above the other three issues. In the other conditions, prioritization of jobs, Medicare, and transportation were similar to here, while prioritization of climate change was indicated by (1) 19% Democrats, 22% Republicans; (2) 19% Democrats, 79% Republicans; and (3) 81% Democrats, 22% Republicans

Participants were asked to suppose that a nationally representative sample of Americans reported their prioritization of policy issues. They viewed one of four infographics that showed the results of this supposed survey for each of our norms conditions (Fig. 1). Democratic high or low prioritization of climate policy was crossed with Republican high or low prioritization of climate policy. For analysis, ingroup and outgroup prioritization of climate policy were coded as either low (– 0.5) or high (+ 0.5), with Democratic prioritization comprising ingroup norms for Democrats and outgroup norms for Republicans, and Republican prioritization comprising ingroup norms for Republicans and outgroup norms for Democrats.

After viewing the infographic, participants reported their personal climate policy prioritization. We operationalized climate policy prioritization with two randomly ordered measures. One measure comprised a 7-point Likert scale on which participants were asked to “Please indicate whether you think dealing with climate change and global warming should be the top priority, an important but lower priority, a not too important priority, or should not be done” on a 7-point scale from *should not be done* (– 3) to *top priority* (+ 3). For the second measure, participants were asked to rank the following randomly-ordered policy issues: “dealing with climate change and global warming,” “improving the job situation,” “improving the country’s roads, bridges, and public transportation systems,” and “taking steps to make the Medicare system more financially sound.” Rank of climate policy was coded from *lowest* (+ 1) to *highest* (+ 4) rank. This second measure was included to prompt participants to make tradeoffs between the issues, to account for the fact that participants could rate multiple issues as high priority in the first measure.

Finally, participants completed a manipulation check. They were asked, “How much does the average Democrat think climate policy should be prioritized?” and “How much does the average Republican think climate policy should be prioritized?” and responded on a 7-point scale from *should not be done* (– 3) to *top priority* (+ 3). There were two open-ended debriefing questions used to assess skepticism about the information presented the study. Participants were asked, “Did you wonder at any point whether there was more than meets the eye to any of the procedures that we had you complete today? That is, do you think that there might have been any information that we held back from explaining from you about the experiment until now?” and then “If you had any suspicions, do you think they affected your behavior during the study?”.

### Results

#### Manipulation check

The manipulations influenced perceived norms as intended. We analyzed estimates of Democrats’ and Republicans’ prioritization of climate policy with 2 (Target: Democrat or Republican) × 2 (Democratic norms: prioritize or do not prioritize) × 2 (Republican norms: prioritize or do not prioritize) ANOVAs including target as a within-subjects factor and Democratic and Republican norms as between-subjects factors. Participants perceived that Democrats prioritized climate policy more in the condition where Democratic norms prioritized it (*M* = 2.04, *SD* = 0.95) than in the condition where they did not (*M* = 0.87, *SD* = 1.56, *F*(1, 135) = 27.83, *p* < .001). This analysis only included Democratic participants’ perceptions of Democratic norms; due to a coding error, Republican participants were not asked this question. Perceptions of Republican prioritization of climate policy were higher in the condition where Republican norms prioritized climate policy (*M* = 0.20, *SD* = 1.89) than in the condition where they did not (*M* = – 1.06, *SD* = 1.39; *F*(1, 215) = 31.38, *p* < .001).

On the debriefing questions, 20 participants expressed doubt about the information provided in the manipulation, including seventeen of the 137 Democrats (including those who leaned toward the Democratic party but did not fully identify with it) and three of the 80 Republicans (test of difference in proportion of doubters between parties: *χ*^2^ = 3.55, *p* = .060. Our key tests remained significant when excluding these 20 participants.

#### Effects of social norms on climate policy prioritization and ranking

**Fig. 2:**
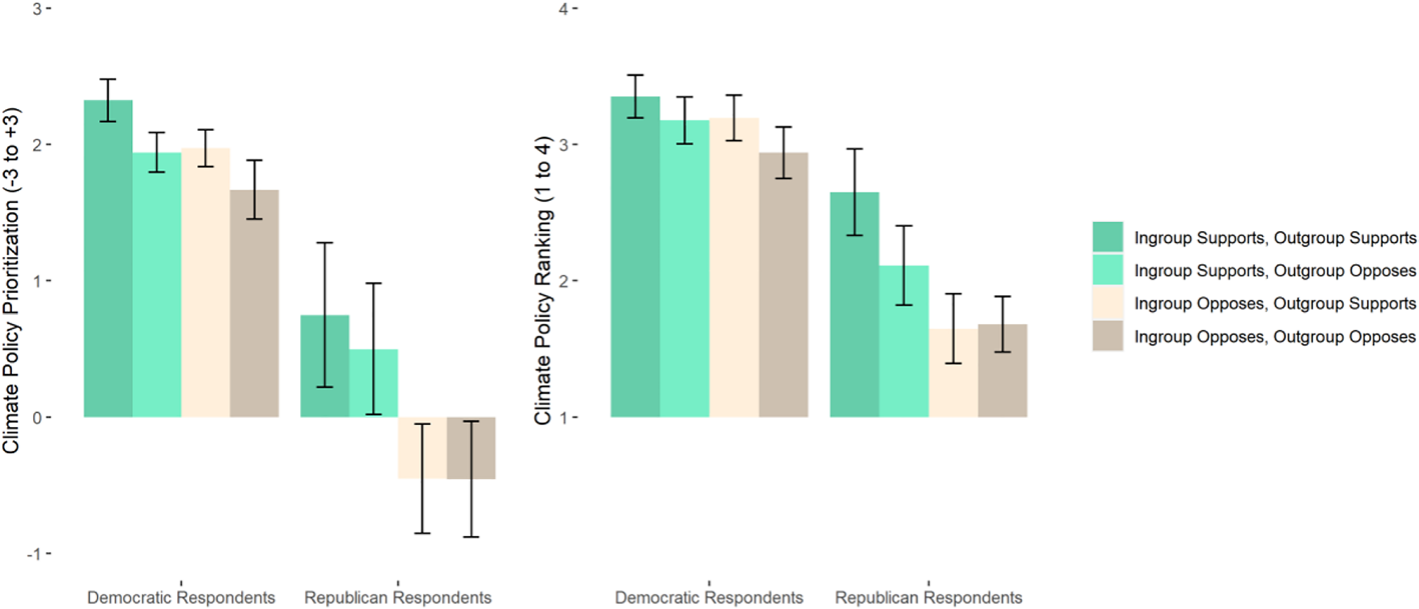
Study 2 observed means of climate policy prioritization, coded on a scale from *should not be done* (– 3) to *top priority* (+ 3), and means of climate policy ranking, coded on a scale from + 1 to + 4, with scores closer to the top of the graph representing higher prioritization and ranks

Confirming our hypotheses, ingroup norms influenced participants’ climate policy prioritization, and the effect of ingroup norms was substantially larger than the effect of outgroup norms (Fig. 2). We submitted each of our two measures of prioritization to a 2 (Ingroup norms: prioritize or do not prioritize) × 2 (Outgroup norms: agree with ingroup or oppose ingroup) × 2 (Partisan identification: Democrat or Republican) ANOVA (Table 3). On the Likert scale measure of policy prioritization, Democrats (*M* = 1.98, *SD* = 0.98) prioritized climate policy more than did Republicans (*M* = 0.06, *SD* = 2.09; *F*(1, 215) = 84.53, η_*p*_^2^ = 0.29, *p* < .001). More important for our hypotheses, participants across both political parties prioritized climate policy more when their ingroup prioritized it (*M* = 1.59, *SD* = 1.65) than when their ingroup did not prioritize it (*M* = 0.96, *SD* = 1.79; *F*(1, 215) = 11.44, η_*p*_^2^ = 0.05, *p* = .001). The effect of the outgroup agreeing with ingroup norms was not significant (*F*(1, 215) = 0.15, η_*p*_^2^ < 0.01, *p* = .695).

**Table 3:** Study 2 ANOVA results predicting prioritization of climate policy and ranking of climate policy

	Prioritization of climate policy			Ranking of climate policy		
	*F*	η_*p*_^2^	*p*	*F*	η_*p*_^2^	*p*
Partisan identification	84.53	0.29	< .001	56.78	0.21	< .001
Ingroup norms	11.44	0.05	.001	9.05	0.04	.003
Outgroup agrees	0.15	< 0.01	.695	0.66	< 0.01	.418
Ingroup * outgroup	1.31	0.01	.253	2.39	0.01	.123
Party * ingroup	3.46	0.02	.064	2.90	0.01	.090
Party * outgroup	0.04	< 0.01	.838	1.14	0.01	.286
Party * ingroup * outgroup	0.28	< 0.01	.599	0.02	< 0.01	.901

The measure of ranking of climate policy yielded a similar pattern of results. Democrats (*M* = 3.17, *SD* = 1.00) ranked climate policy higher than did Republicans (*M* = 2.01, *SD* = 1.24; *F*(1, 215) = 56.78, η_*p*_^2^ = 0.21, *p* < .001). Participants across both political parties ranked climate policy higher when their political ingroup prioritized climate policy (*M* = 2.95, *SD* = 1.18) than when their ingroup did not prioritize it (*M* = 2.54, *SD* = 1.24; *F*(1, 215) = 9.05, η_*p*_^2^ = 0.04, *p* = .003). Whether outgroup norms agreed with ingroup norms did not significantly influence policy support. (*F*(1, 215) = 0.66, η_*p*_^2^ < 0.01, *p* = .418).

We explored whether ingroup and outgroup norms differentially affected Democrats and Republicans. The sample is underpowered to examine such interaction effects, so these tests should be interpreted with caution. On the Likert measure of prioritization, although ingroup norms did not significantly interact with partisan identification (*F*(1, 215) = 3.46, η_*p*_^2^ = 0.02, *p* = .064), the effect of ingroup norms was significant among Republican participants (*F*(1, 215) = 10.74, η_*p*_^2^ = 0.05, *p* = .001) but not Democratic participants (*F*(1, 215) = 1.58, η_*p*_^2^ = 0.01, *p* = .211). There was no interaction between ingroup norms and whether the outgroup aligned with the ingroup among either Republican participants (*F*(1, 215) = 0.15, η_*p*_^2^ < 0.01, *p* = .697) or Democratic participants (*F*(1, 215) = 1.90, η_*p*_^2^ = 0.01, *p* = .169).

On the ranking measure of prioritization, although the effect of ingroup norms did not significantly interact with participant partisan identification (*F*(1, 215) = 2.90, η_*p*_^2^ = 0.01, *p* = .090), the effect was significant among Republican participants (*F*(1, 215) = 7.79, η_*p*_^2^ = 0.04, *p* = .003) but not Democratic participants (*F*(1, 215) = 0.08, η_*p*_^2^ = 0.01, *p* = .283). There was no interaction between ingroup norms and whether the outgroup aligned with the ingroup among Republican participants (*F*(1, 215) = 1.10, η_*p*_^2^ = 0.01, *p* = .294) or Democratic participants (*F*(1, 215) = 1.37, η_*p*_^2^ = 0.01, *p* = .242).

We conducted an exploratory analysis to examine whether personal prioritization of climate policy was higher in the condition with bipartisan norms of prioritization of climate policy. This analysis was motivated by the practical importance of bipartisan prioritization of climate policy. If either party were dissuaded from prioritizing climate policy when the other party prioritized it, the possibility for bipartisan prioritization outside of the lab would be hindered. We used a set of orthogonal contrast codes to test this comparison, along with a comparison between the condition in which neither party prioritized climate policy and the conditions in which only one party prioritized it, and a comparison between the two conditions in which one party prioritized it. Bipartisan norms of support resulted in higher personal prioritization on the Likert measure (*M* = 1.74, *SD* = 1.76) than the average of the other conditions (*M* = 1.12, *SD* = 1.72; *F*(1, 215) = 8.08, η_*p*_^2^ = 0.04, *p* = .005). Bipartisan support also resulted in higher ranking (*M* = 3.09, *SD* = 1.27) than the average of the other conditions (*M* = 2.63, *SD* = 1.23; *F*(1, 215) = 9.61, η_*p*_^2^ = 0.04, *p* = .002).

There were no significant differences overall between the condition with bipartisan opposition and the two conditions with one party or the other prioritizing climate policy on personal prioritization (*F*(1, 215) = 2.39, η_*p*_^2^ = 0.01, *p* = .123) or ranking (*F*(1, 215) = 1.47, η_*p*_^2^ = 0.01, *p* = .227). There were also no significant differences in policy prioritization between the condition in which Democrats prioritized climate policy and Republicans did not and the condition in which Republicans prioritized it and Democrats did not (*F*(1, 215) = 2.44, η_*p*_^2^ = 0.01, *p* =.120). Similarly, there were no differences in policy ranking on average between the condition in which Democrats prioritized climate policy and Republicans did not and the condition in which Republicans prioritized it and Democrats did not (*F*(1, 215) = 1.04, η_*p*_^2^ = 0.01, *p* = .308).

### Discussion

Through experimental manipulations, we demonstrated that political ingroup norms influence personal prioritization of climate policy. The effect was smaller and not significant for outgroup norms. Accordingly, the presence of norms of prioritization of climate policy within both parties leads to the highest average personal prioritization of people across both parties. We also found directional but nonsignificant interactions suggesting the influence of ingroup norms is larger for Republicans than for Democrats. Fully powered tests of these interactions may demonstrate significant effects.

Given that ingroup norms affected policy prioritization across the full sample, and that this effect was greater among Republican participants than Democrats (although nonsignificant), we can conclude that Republicans prioritize climate policy more when they perceive that other Republicans also prioritize climate policy—a practically important finding given that Republicans place lower priority on climate policy than Democrats do (Funk and Kennedy 2020; Pew Research Center 2017, 2021). That norms about climate policy prioritization are more influential among Republicans than Democrats is consistent with research demonstrating that norms have stronger effects on behaviors that are less important to individuals’ identities (Cialdini and Jacobson 2021).

## General discussion

Social norms predict and influence public prioritization of climate policy. In Study 1, people’s prioritization of climate policy was most strongly predicted by beliefs about fellow partisans’ priorization of climate policy. Norms of the political ingroup were a stronger predictor of personal climate policy prioritization than personal belief in climate change, political orientation, environmental identity, environmental values, and demographic factors. In Study 2, in an experiment, ingroup social norms influenced personal climate policy prioritization whereas outgroup norms did not. Average prioritization across the full sample was highest when we told participants that it was normative within both parties to prioritize climate policy. Together, these findings highlight the importance of social norms for climate policy prioritization. They also suggest that public bipartisan prioritization of climate policy is possible outside of the lab, given that people did not react negatively against the outgroup prioritizing climate policy.

### Theoretical implications

This work replicates and extends previous work on the influence of social norms (Miller and Prentice 2016; Tankard and Paluck 2016) to the domain of climate policy prioritization. While prior work established the importance of norms to environmental policy support (Alló and Loureiro 2014; De Groot and Schuitema 2012; Rinscheid et al. 2021), prioritization of a policy issue is distinct from support for a specific policy and is a new, important outcome.

The present results also contribute to the comparison of ingroup and outgroup influence. Prior research shows that people both assimilate to their ingroups and differentiate from outgroups (Berger and Heath 2008; Chan et al. 2012; Ehret et al. 2018; Iyengar et al. 1999; Mummendey and Wenzel 1999). Negative reactions to outgroups might be especially influential in highly polarized contexts such as climate policy in the United States, where Democrats and Republicans actively distrust and dislike each other (Druckman et al. 2021; Finkel et al. 2020; Flores et al. 2022; Iyengar et al. 2019; Iyengar and Westwood, 2015; Skitka 2010). However, our findings demonstrate that people’s prioritization of climate policy is more positively influenced by ingroup norms than it is negatively influenced by outgroup norms, extending previous work about partisan norms in the context of climate policy (Ehret et al. 2018; Van Boven et al. 2018). In other words, Republicans are not highly sensitive to what Democrats think, and Democrats are not highly sensitive to what Republicans think.

It is also theoretically meaningful that ingroup norms predict climate policy prioritization over and above individual differences and demographic characteristics. Much work in environmental psychology has examined individual-level characteristics such as personal beliefs, attitudes, ideologies, values, and demographics as predictors and causes of environmental attitudes and behavior (e.g., Campbell and Kay 2014; Dunlap and McCright 2008). Our work suggests that social factors are at least, if not more, important than individual factors in the prioritization (or deprioritization) of climate policy, aligning with a recent movement within the field of environmental psychology to focus more on group-level factors (Masson and Fritsche 2021; Pearson et al. 2016).

Study 1 was conducted in the period of time leading up to the 2016 presidential election, which might have been a particularly polarized time within the country. This raises the question of the generalizability of these findings. However, similar patterns of partisan social norms and polarized policy opinion between political parties were found in a study conducted in 2014 (Van Boven et al. 2018). Study 2 was conducted in 2019, 2 years after the 2016 presidential election and not during a presidential election cycle. Presumably, if polarization were ever to decrease, it would be when presidential elections were distant in time. However, climate change was still highly polarized and people were still strongly influenced by partisan norms at the time of Study 2.

### Practical implications

Successful enactment of climate policy in the United States is critical to the mitigation of climate change. Most of the United States believes in climate change (Marlon et al. 2020; Van Boven et al. 2018), yet the country has not implemented a federal climate policy. Why? Much research has emphasized skepticism about climate change. Although it is true that some (powerful) stakeholders are resistant to accepting that climate change is happening, we emphasize that social influence may be a particularly limiting factor, causing the deprioritization of climate change even among those who believe in and are concerned about climate change. Prioritization of climate policy is distinct from belief in climate change, and social norms influence policy priorization more so than belief in climate change does. Public prioritization can be motivated by bipartisan social norms of prioritization. And, policymakers are influenced by public opinion (Jones and Baumgartner 2005; Stimson et al. 1995), so social norms could be a promising lever to bring the federal government closer to enacting climate policy.

Norms within political parties may not presently reflect prioritization of climate policy (Pew Research Center 2021), especially within the Republican party (Kennedy and Johnson 2020). To the extent that others are perceived as considering climate change a low priority, these social norms may contribute to a vicious cycle of continuous deprioritization of climate policy. However, our results indicate a pathway to improve this. Evolving norms of positive attitudes toward climate change solutions can improve individuals’ own attitudes, which in turn can reinforce positive norms (Sparkman et al. 2021). Advocates of climate policy should identify the broad and emerging areas of consensus where and when people are prioritizing climate change and amplify those, to increase perceptions that it is normative to prioritize government action on climate change. For example, climate change communications could emphasize subgroups that prioritize climate policy, such as the 81% of moderate Republicans that support tax credits for businesses that develop carbon capture and storage and the 76% of moderate Republicans that support restrictions on power plant emissions (Tyson 2021). Communications can also emphasize aspects of climate change and types of climate policies that are more highly prioritized. One public opinion poll reported that “clean energy” was prioritized substantially higher than “global warming,” among both Democrats and Republicans (Leiserowitz et al. 2021). If the goal is to foster enactment of climate policy, it will behoove agents of behavior change to appreciate the importance of and leverage the power of social norms.

## Supplementary Information

Below is the link to the electronic supplementary material.

Supplementary file1 (DOCX 311 KB)

## Data Availability

All data, materials, and code are available at https://osf.io/mf945/?view_only=f763f59f7fac4cdf9ee972fc046cc736.

## Footnotes

1 A similar pattern of results emerged when using the raw 4-point priority ratings instead of the *z*-scores. Perceived ingroup policy prioritization significantly predicted individual policy prioritization (*b* = 0.27, *SE* = 0.03, η_*p*_^2^ = 0.10, *p* < .001). Perceived outgroup policy prioritization predicted personal prioritization, though less so than ingroup norms (*b* = 0.08, *SE* = 0.03, η_*p*_^2^ = 0.01, *p* = .006). Model fit improved significantly when including the social norms predictors in the model (*F*(2, 795) = 48.93, *p* < .001).

2 In this control condition, respondents read that both parties prioritized climate change equally to other policy issues. We did not include this condition in the present analyses because its purpose was to compare whether the positive influence of a party’s prioritization was symmetrical to the negative influence of a party’s de-prioritization. This control condition was not needed to compare ingroup and outgroup influence.
