# Supplementary material for: Social norms explain prioritization of climate policy
Source: Clim Change. 2022 Jul 18;173(1-2):10. doi: 10.1007/s10584-022-03396-x (PMC9289929; doi:10.1007/s10584-022-03396-x)
Supplement: Supplementary file 1 — Supplementary file1 (DOCX 311 KB) [file 10584_2022_3396_MOESM1_ESM.docx]

**Electronic Supplementary Material**

**Study 1 Survey Text**

Please indicate how much you agree with the following statements (strongly disagree, moderately disagree, slightly disagree, neither agree nor disagree, moderately agree, strongly agree):

1. I am concerned with environmental issues.
2. I am an environmentalist.

Do you agree or disagree that (strongly disagree, mildly disagree, unsure, mildly agree, strongly agree):

1. We are approaching the limit of the number of people the earth can support.
2. Humans have the right to modify the natural environment to suit their needs.
3. When humans interfere with nature it often produces disastrous consequences.
4. Human ingenuity will insure that we do NOT make the earth unlivable.
5. Humans are severely abusing the environment.
6. The earth has plenty of natural resources if we just learn how to develop them.
7. Plants and animals have as much right as humans to exist.
8. The balance of nature is strong enough to cope with the impacts of modern industrial nations.
9. Despite our special abilities humans are still subject to the laws of nature.
10. The so-called "ecological crisis" facing humankind has been greatly exaggerated.
11. The earth is like a spaceship with very limited room and resources.
12. Humans were meant to rule over the rest of nature.
13. The balance of nature is very delicate and easily upset.
14. Humans will eventually learn enough about how nature works to be able to control it.
15. If things continue on their present course, we will soon experience a major ecological catastrophe.

Do you typically identify yourself as a Democrat, Republican, Independent, or what?

- Democrat
- Republican
- Independent
- Other (please specify in text box)
- No preference

*If they answered Democrat above:* Would you call yourself a strong Democrat or a not very strong Democrat?

- Strong Democrat
- Not Very Strong Democrat

*If they answered Republican above:* Would you call yourself a strong Republican or a not very strong Republican?

- Strong Republican
- Not Very Strong Republican

*If they answered Independent, Other, or No preference above:* Do you think of yourself as closer to the Republican Party or closer to the Democratic party?

- Closer to the Democratic Party
- Closer to the Republican Party
- Neither

For each of the items below please indicate which option best describes you (very liberal, liberal, somewhat liberal, moderate, somewhat conservative, conservative, very conservative):

1. Politically, I consider myself
2. On economic issues, I consider myself
3. On social issues, I consider myself

What is your gender?

- Male
- Female
- Other

How old are you? (Leave blank if you prefer not to say.)

Which racial or ethnic group best describes you?

- Asian; Asian-American (please specify)
- Black; African-American
- Hispanic; Latino-American (please specify)
- Native American
- Native Pacific Islander
- White; Caucasian-American
- Other (please specify)

Please indicate the highest level of education completed.

- Grammar School
- High School or equivalent
- Vocational/Technical School (2 year)
- Some College
- College Graduate (4 years)
- Master’s Degree
- Doctoral Degree
- Professional Degree (MD, JD, etc.)
- Other (please specify)

Please indicate your current household income in U.S. dollars.

- Rather not say
- Under $10,000
- $10,000-$19,999
- $20,000-$29,999
- $30,000-$39,999
- $40,000-$49,999
- $50,000-$74,999
- $75,000-$99,999
- $100,000-$150,000
- Over $150,000

Global warming refers to the recent and ongoing rise in global average temperature near the Earth's surface. Increasing concentrations of greenhouse gasses is the primary cause of global warming. Global warming, in turn, is causing climate patterns to change. Climate change includes major changes in temperature, precipitation, wind patterns, or other effects that occur over several decades or longer. Please indicate how much you agree with the following statements (strongly disagree, moderately disagree, slightly disagree, neither agree nor disagree, slightly agree, moderately agree, strongly agree).

1. Climate change is happening.
2. Climate change poses a risk to human health, safety, and prosperity.
3. Human activity is largely responsible for recent climate change.
4. Reducing greenhouse gas emissions will reduce global warming and climate change.

*Responding to the same four items as the previous question:*

Consider how the average Democrat/Republican would answer the following questions. Please indicate how much you believe the average Democrat agrees with the following statements.

We'd like to ask you about priorities for the next President and Congress in the coming years. Please read each item from list below, indicating whether it should be top priority, important but lower priority, not too important, or should it not be done.

1. Dealing with climate change and global warming
2. Strengthening the nation’s economy
3. Improving the job situation
4. Defending the country from terrorist attacks
5. Improving the educational system
6. Taking steps to make the Social Security system financially sound
7. Reducing the budget deficit
8. Taking steps to make the Medicare system financially sound
9. Reducing health care costs
10. Reforming the nation’s tax system
11. Reducing crime
12. Dealing with the problems of poor and needy people
13. Protecting the environment
14. Dealing with global trade issues
15. Improving the country’s roads, bridges, and public transportation systems
16. Dealing with the issue of illegal immigration
17. Reducing the influence of lobbyists and special interest groups in Washington
18. Strengthening the U.S. Military
19. Dealing with the nation’s energy problem

*Responding to the same policy items as the previous question:*

Consider how the average Democrat would respond to the following statements, which are the same ones you read earlier. How do you think the average Democrat would respond? Please read each item from list below, and indicate what the average Democrat believes should be top priority, important but lower priority, not too important, or should it not be done.

Respondents also reported on a variety of other measures, including preference for pain, big five personality characteristics, locomotion, and happiness, among others.

**ESM Table 1**

***Demographic information for Study 1.***

|  |  | Number | Percent |
| --- | --- | --- | --- |
| *Partisan Identification* | Democrat | 463 | 52.20% |
|  | Republican | 424 | 47.80% |
| *Gender* | Male | 436 | 49.21% |
|  | Female | 445 | 50.23% |
|  | Other | 5 | 0.56% |
| *Income* | Decline to say | 28 | 3.16% |
|  | < $10,000 | 50 | 5.65% |
|  | $10,000-$19,999 | 88 | 9.94% |
|  | $20,000-$29,999 | 102 | 11.53% |
|  | $30,000-$39,999 | 79 | 8.93% |
|  | $40,000-$49,999 | 96 | 10.85% |
|  | $50,000-$74,999 | 150 | 16.95% |
|  | $75,000-$99,999 | 120 | 13.56% |
|  | $100,000-$150,000 | 95 | 10.73% |
|  | >$150,000 | 77 | 8.70% |
| *Highest Level of Education* | Grammar School | 27 | 3.05% |
|  | High School/Equivalent | 261 | 29.49% |
|  | Vocational/Technical School | 49 | 5.54% |
|  | Some College | 194 | 21.92% |
|  | College Graduate | 226 | 25.54% |
|  | Master’s Degree | 93 | 10.51% |
|  | Doctoral Degree | 14 | 1.58% |
|  | Professional Degree (JD, MD, etc.) | 15 | 1.69% |
|  | Other | 6 | 0.68% |
| *Ethnicity* | Asian, Asian-American | 35 | 3.95% |
|  | Black, African-American | 103 | 11.64% |
|  | Hispanic, Latino-American | 132 | 14.92% |
|  | Native American | 4 | 0.45% |
|  | Native Pacific Islander | 2 | 0.23% |
|  | White, Caucasian-American | 604 | 68.25% |
|  | Other | 5 | 0.56% |

**Study 1 Models Predicting Belief in Climate Change**

We ran similar models predicting belief in climate change as we report predicting prioritization of climate policy (ESM Table 2).

**ESM Table 2**

**Regression models with unstandardized coefficients for Study 1 predicting belief in climate change by norms and individual differences.**

|  | **Model 1** | | | **Model 2** | | |
| --- | --- | --- | --- | --- | --- | --- |
|  | ***b*** | ***SE*** | **η*_p_*^2^** | ***b*** | ***SE*** | **η*_p_*^2^** |
| Intercept | 1.32*** | 0.04 | 0.53 | 1.32*** | 0.04 | 0.63 |
| Social Norms | | | | | | |
| Ingroup Climate Change Belief |  |  |  | 0.46*** | 0.02 | 0.33 |
| Outgroup Climate Change Belief |  |  |  | 0.06*** | 0.02 | 0.02 |
| Values, Ideology and Identity | | | | | | |
| Political Orientation | –0.15*** | 0.02 | 0.05 | –0.05* | 0.02 | 0.01 |
| Environmental Identity | 0.33*** | 0.02 | 0.20 | 0.20*** | 0.02 | 0.10 |
| Environmental Values (NEP) | 1.00*** | 0.07 | 0.23 | 0.77*** | 0.06 | 0.19 |
| Demographics | | | | | | |
| Age | –0.01* | < 0.01 | 0.01 | < 0.01* | < 0.01 | 0.01 |
| Income | < 0.01 | 0.02 | < 0.01 | –0.01 | 0.01 | < 0.01 |
| Gender | < 0.01 | 0.04 | < 0.01 | 0.01 | 0.03 | < 0,01 |
| Education | 0.03 | 0.03 | < 0.01 | 0.04 | 0.02 | < 0.01 |
| Black | 0.06 | 0.12 | < 0.01 | 0.02 | 0.10 | < 0.01 |
| Hispanic | < 0.01 | 0.11 | < 0.01 | 0.07 | 0.09 | < 0.01 |
| Other Minority | –0.01 | 0.16 | < 0.01 | –0.08 | 0.13 | < 0.01 |
| R^2^ |  | .57 |  |  | .71 |  |

Note. *** indicates p < .001; ** indicates p < .01; * indicates p < .05. Sample sizes vary due to missing data (Model 1: *N* = 821; Model 2: *N* = 687). Coefficients are unstandardized. Belief in climate change, environmental identity, environmental values, age, income, and education are continuous. Higher scores on political orientation indicate being more conservative and Republican. Gender is coded as male (–1) and female (+1). Black, Hispanic or Latino, and Other Minority are dummy coded with White as the reference group.

**Study 2 Infographics for the Experimental Manipulation**

Below are the four infographics comprising the experimental manipulation, each representing one of the conditions.


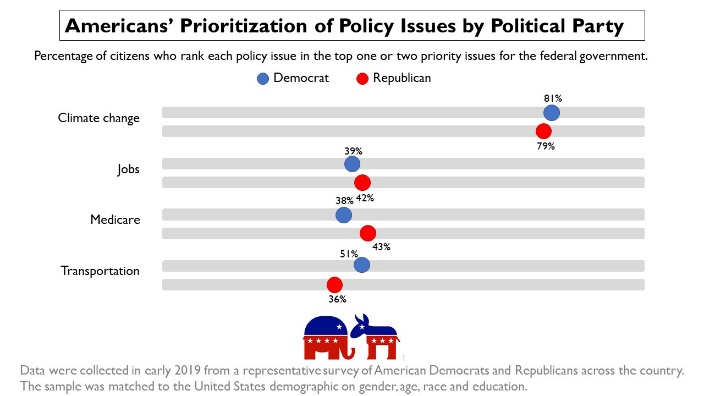

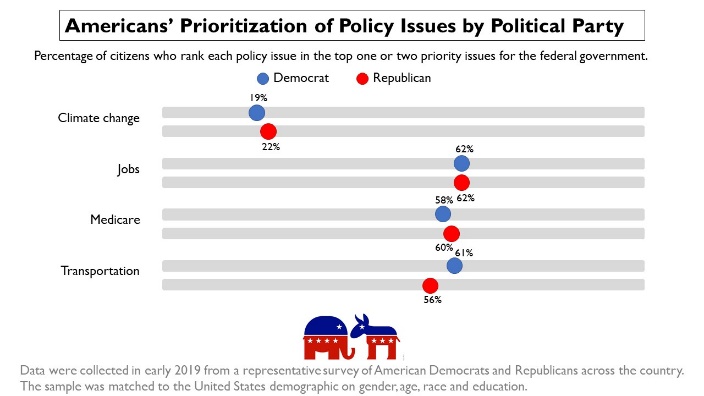

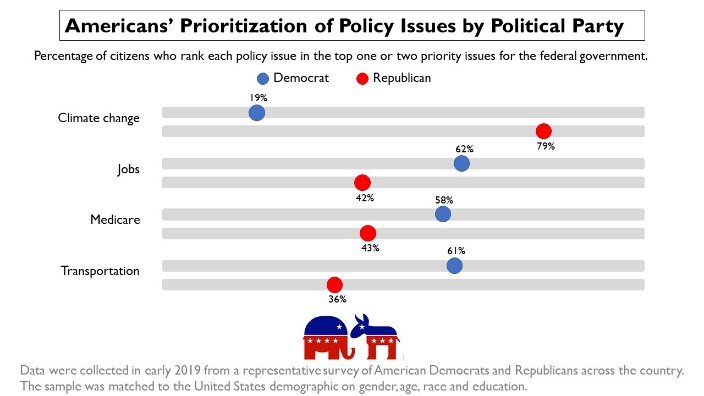

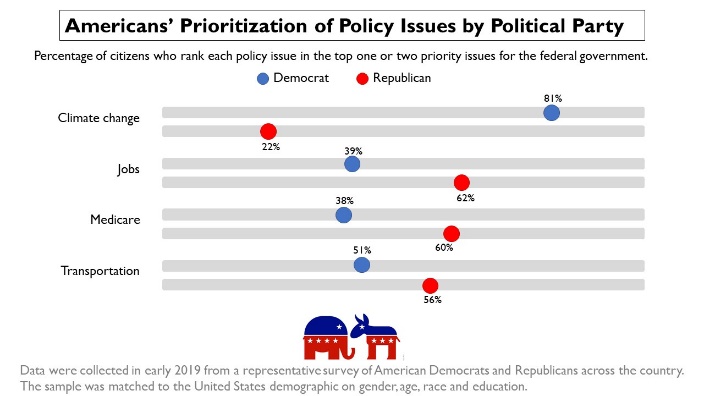


**Study 2 Cell Sizes**

**ESM Table 3**

***Cell sizes within the 2 (Ingroup norms: high or low priority) × 2 (Outgroup norms: high or low priority) × 2 (Respondent partisan identification: Democrat or Republican) experimental design of Study 2. We used Qualtrics’ randomization feature to evenly distribute participants across conditions.***

|  | Ingroup Supports | | Ingroup Opposes | |
| --- | --- | --- | --- | --- |
|  | Outgroup Supports | Outgroup Opposes | Outgroup Supports | Outgroup Opposes |
| Democrat | 34 | 34 | 36 | 33 |
| Republican | 20 | 18 | 20 | 22 |

**Identifying Policy Issues for Study 2**

We chose the three other issues included in the ranking dependent variable in Study 2 based on data from Study 1. We sought to maximize the extent to which the results of our manipulation were due to climate policy prioritization norms and not to partisan differences in opinion about the other three policy issues, so we chose issues that had minimal partisan differences in prioritization. The issues were ranked on the 4-point scale from *should not be done* (+1) to *top priority* (+4). The mean prioritization for “improving the job situation” for Democrats in Study 1 was 3.43 (*SD* = 0.66), with a mean of 3.41 (*SD* = 0.72) for Republicans. For “improving the country’s roads, bridges, and public transportation systems,” the mean score for Democrats was 3.16 (*SD* = 0.77) and for Republicans was 3.13 (*SD* = 0.72). The mean for Democrats in response to “taking steps to make the Medicare system more financially sound” was 3.39 (*SD* = 0.70), which was the same average as for Republicans (*M* = 3.39, *SD* = 0.75).

**ANOVA Tables for Study 2**

**ESM Table 3**

***Study 2 ANOVA results predicting prioritization of climate policy.***

| Predictor | *b* [95% confidence interval] | η*_p_*^2^ | *p* |
| --- | --- | --- | --- |
| Intercept | 1.03 [0.83, 1.23] | 0.33 | < .001 |
| Partisan Identification | –1.89 [–2.30, –1.48] | 0.29 | < .001 |
| Ingroup Norms | 0.70 [0.29, 1.10] | 0.05 | .001 |
| Outgroup Norms | 0.24 [–0.17, 0.64] | 0.01 | .253 |
| Party * Ingroup | 0.76 [–0.05, 1.58] | < 0.01 | .695 |
| Party * Outgroup | –0.22 [–1.03, 0.59] | 0.02 | .064 |
| Ingroup * Outgroup | 0.16 [–0.65, 0.97] | < 0.01 | .599 |
| Party * Ingroup * Outgroup | 0.17 [–1.45, 1.79] | < 0.01 | .838 |

**ESM Table 4**

***Study 2 ANOVA results predicting ranking of climate policy.***

| Predictor | *b* [95% confidence interval] | η*_p_*^2^ | *p* |
| --- | --- | --- | --- |
| Intercept | 0.10 [–0.24, 0.06] | 0.01 | .214 |
| Partisan Identification | –1.14 [0.84, 1.44] | 0.21 | < .001 |
| Ingroup Norms | 0.46 [–0.76, –0.16] | 0.04 | .003 |
| Outgroup Norms | 0.24 [–0.53, 0.06] | 0.01 | .123 |
| Party * Ingroup | 0.52 [–1.12, 0.08] | < 0.01 | .418 |
| Party * Outgroup | 0.04 [–0.64, 0.56] | 0.01 | .090 |
| Ingroup * Outgroup | 0.25 [–0.84, 0.35] | < 0.01 | .901 |
| Party * Ingroup * Outgroup | 0.65 [–1.85, 0.55] | 0.01 | .286 |

**Partisan Differences in the Effects of Social Norms**

***Study 1***

We added interactions both between ingroup norms and outgroup norms of belief in climate change and prioritization of climate policy with partisan identification to the model predicting personal climate policy prioritization from norms, individual difference characteristics and demographics. Significant interactions would indicate that norms are more important to one party than the other. Ingroup norms interacted with political orientation (*b* = 0.06, *SE* = 0.02, η*_p_*^2^ = 0.01, *p* = .008), such that the effect of norms was stronger for more conservative and Republican participants, though with a small effect size. Outgroup norms did not interact with political orientation (*b* = –0.03, *SE* = 0.02, η*_p_*^2^ < 0.01, *p* = .530).

***Study 2***

Partisan identification did not interact with ingroup norms condition or outgroup norms condition in the models predicting either climate policy prioritization or climate policy ranking, as shown in ESM Tables 3 and ESM 4.

Across both studies, we did not find strong evidence that norms mattered more for one political party or the other, contributing to the literature that argues the existence of bias on both sides of the political aisle. Some existing literature argues that Democrats or liberals are not equally biased with Republicans or conservatives (Baron & Jost, 2019; Brady et al., 2019; Jost, 2017). Other work argues that bias exists within both parties and ideologies, albeit sometimes through different processes (Brandt et al., 2014; Conway et al., 2016; Crawford, 2017; Ditto et al., 2019; Washburn & Skitka, 2018). We found that ingroup social norms were more predictive of personal prioritization of climate policy for Republicans than for Democrats, but found no evidence of partisan differences in the role of norms in belief in climate change in Study 1 or in the effect of norms on either climate policy prioritization measure in Study 2. Largely our results showed that norms are equally important to Democrats’ and Republicans’ personal climate policy prioritization.
